# Supplementary material for: Acceleration-Based Estimation of Vertical Ground Reaction Forces during Running: A Comparison of Methods across Running Speeds, Surfaces, and Foot Strike Patterns
Source: Sensors (Basel). 2023 Oct 25;23(21):8719. doi: 10.3390/s23218719 (PMC10648662; doi:10.3390/s23218719)
Supplement: Supplementary file 1 [file sensors-23-08719-s001.zip › sensors-2625835-supplementary.pdf]

Supplementary Materials

for

Acceleration-based estimation of vertical ground reaction forces during running:

A comparison of methods across running speeds, surfaces, and foot strike patterns

Sections

SA. IMU processing details
SB. Description of force estimation methods and their implementation
SC. Results across wearable, segment, and tilt-corrected coordinate systems

SA. IMU processing details

A brief overview of our IMU data processing is provided in the main text. Here, we expand on the processing details. After collecting raw data from each IMU, data were downloaded and processed offline using the following steps:

SA.1. Calibration;

SA.2. Quiet period identification;

SA.3. Bias removal;

SA.4. Saturation correction;

SA.5. Low-pass filtering;

SA.6. Drift correction;

SA.7. Orientation estimation and gravity subtraction;

SA.8. Coordinate system transformation.

After these processing steps, each of the 642 trials we analyzed was segmented using a 10 N threshold from the time synchronized force signal. Trials were then entered into each of the individual methods as an f × 4 matrix (with f rows representing the number of frames during stance and the four columns representing time stamps and three acceleration axes).

SA.1. Calibration

All IMU data collected during this experiment were corrected with IMU-specific calibration matrices. These matrices were calculated by conducting a calibration procedure that ensured each IMU accurately expressed accelerations and angular velocities in an orthogonal coordinate system that was oriented square to the IMU housing.

To do so, each IMU was secured to a centrifuge (ClearPath MCVC, Teknic, Victor, NY, USA) with custom 3D printed jigs (SOLIDWORKS 2019, Dassault Systèmes, Vélizy-Villacoublay, France) and calibrated in six orientations at 16 known accelerations (from 0 to 41.42 g where 1 g = 9.8 m/s^2 [104,105]) and angular velocities (from 0 to 78.54 rad/s). Adapting methods from Coolbaugh et al. [81], known data (K) from the centrifuge and measured data (M) from the IMU were used to calculate

3 × 7 calibration matrices for each IMU (C; three signed magnitude terms, three absolute magnitude terms, and one bias term per axis) and quantify the sensor accuracy and precision with a hold-back procedure after subtracting out the biases observed during a quiet period (B).

$$C * (M + B) = K \quad \text{Equation S1}$$

One potential limitation of this procedure is that it treats each triaxial sensor independently (primary accelerometer, secondary accelerometer, and gyroscope) and assumes that their values do not affect each other. This assumption was tested while piloting the calibration procedure by quantifying inter-sensor dependencies between the primary accelerometer and gyroscope and between the secondary accelerometer and gyroscope. Observed dependencies were negligible and independent sensor calibration matrices yielded the best results; thus, we felt confident using this approach (which avoids the indeterminacy of the primary and secondary accelerometer having the same K values).

After calibration, the IMU primary accelerometer errors were ≤ 0.01 ± 0.04 g, secondary accelerometer errors were ≤ 0.05 ± 0.07 g, and gyroscope errors were ≤ 0.01 ± 0.01 rad/s.

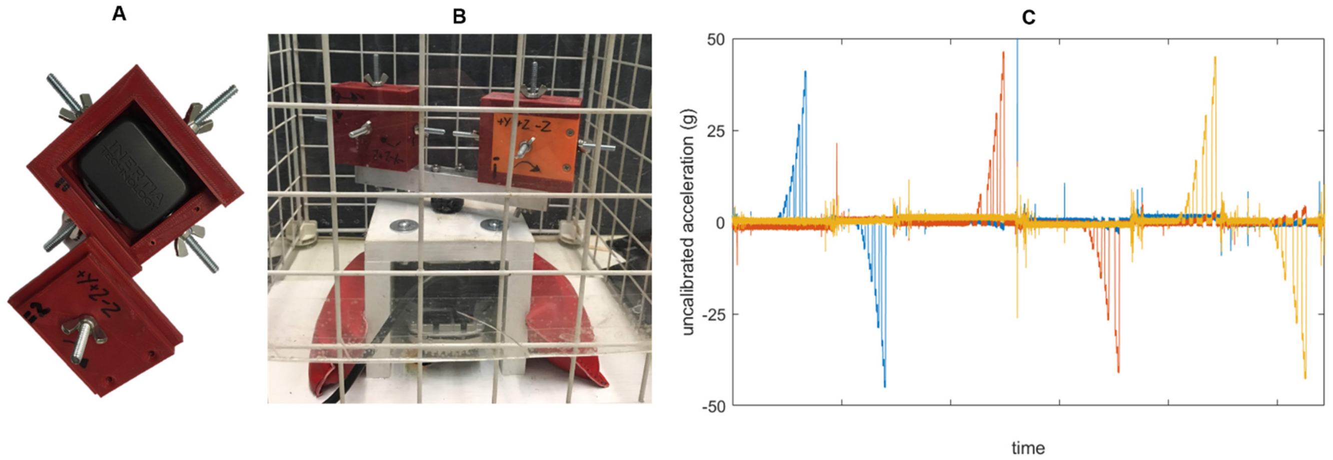

Figure S1: (A) An IMU in a 3D printed housing. Computer-aided design software was used to ensure that the IMUs were friction fit square to their housing. **(B)** Two IMUs in their 3D printed housings mounted on the centrifuge. IMUs were checked to be square with an engineer's square and level with a bullseye level. **(C)** Example of measured triaxial accelerations for the secondary accelerometer (M). The 16 accelerations being applied to the IMU in each of the six orientations correspond to known (K) values from the centrifuge. Accelerations between each orientation correspond to the IMU being repositioned on the centrifuge and checked to be square and level.

SA.2. Quiet period identification

Quiet periods were identified throughout the data collection (e.g., participant resting, participant preparing at the start of the runway, participant standing while receiving instruction) and used to periodically check for changes in bias (as bias can vary with battery life and temperature) and reset orientation algorithms (as orientation estimates are prone to drift over prolonged periods; discussed further below). These quiet periods were defined as any period where…

$$\omega_{resultant} < 0.5\, rad/s \quad \text{Equation S2}$$

$$j_{resultant} < 0.01\, m/s^3 \quad \text{Equation S3}$$

…for at least 100 ms.

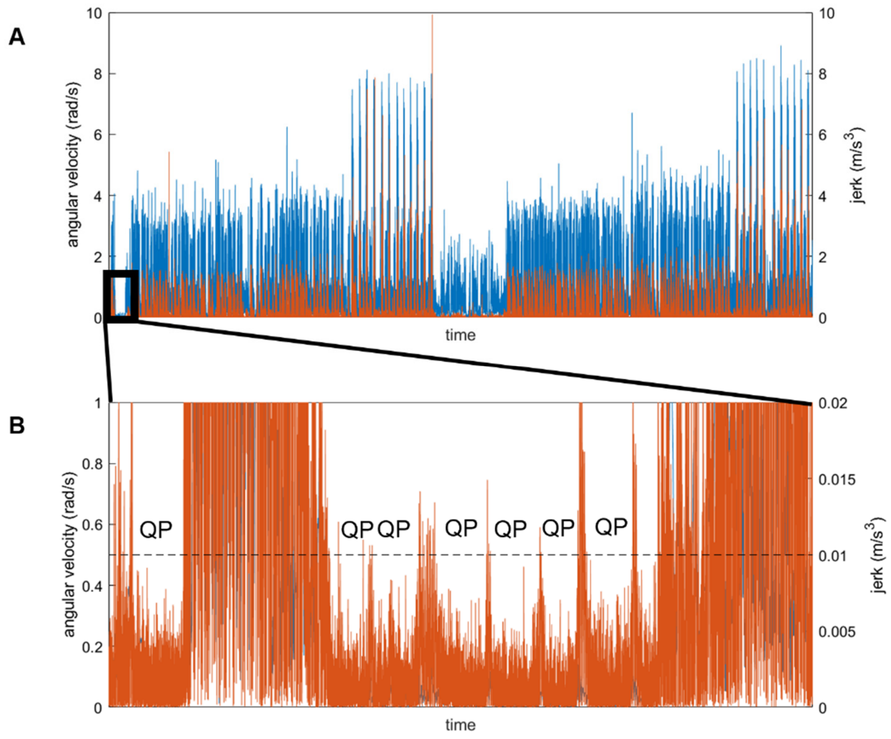

Figure S2: (A) Resultant angular velocity (blue) and jerk (orange) at the sacrum across an entire data collection for a randomly selected example participant. **(B)** Zoomed in to show quiet periods (QP) where resultant angular velocities are < 0.5 rad/s and resultant jerks are < 0.01 m/s^3 for at least 100 ms. Thresholds noted with the dashed horizontal line.

SA.3. Bias removal

When the IMU is quiet, we know that it is not accelerating or rotating and thus, the only thing loading the axes should be the gravity vector. Based on this knowledge, we can create a temporary inertial coordinate system based on gravity:

$$Y = \frac{\sum_{first\ quiet\ frame}^{last\ quiet\ frame} a_{calibrated}}{n_{quiet\ frames}} \qquad \text{Equation S4}$$

We can express Y as a unit vector, then make X and Z orthogonal unit vectors (with arbitrary sense). Using these vectors, we can create a temporary rotation matrix that will align our data with gravity:

$$R_{temp} = \begin{bmatrix} X \\ Y \\ Z \end{bmatrix} \qquad \text{Equation S5}$$

We can then express our data in this temporary inertial coordinate system (and given our calibration, we know that the axes of the accelerometer and gyroscope are exactly aligned so the same rotation matrix can be used for both):

$$a_{temp} = R_{temp} * a_{calibrated} \qquad \text{Equation S6}$$

Given the IMU is quiet, in every frame a_{temp} and ω_{temp} should now equal [0 1 0] g and [0 0 0] rad/s, respectively. Thus, we can calculate bias (B) in the acceleration and angular velocity as the average deviation from those values across the quiet period:

$$B_a = \frac{\sum_{first\ quiet\ frame}^{last\ quiet\ frame}(a_{temp} - [0\ 1\ 0])}{n_{quiet\ frames}}$$ Equation S7

We can then remove the bias and re-express our data in the original coordinates:

$$a_{debias} = a_{calibrated} - R_{temp}^{-1} B_a$$ Equation S8

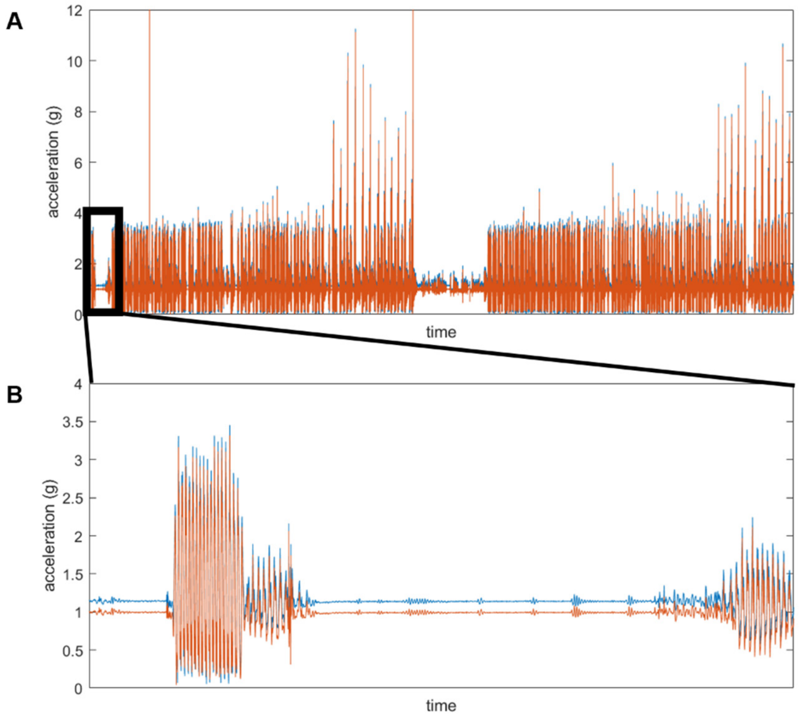

Figure S3: **(A)** Uncorrected resultant sacral acceleration (blue) and de-biased resultant sacral acceleration (orange) across the entire data collection for the randomly selected participant. **(B)** Zoomed in to show that the uncorrected resultant acceleration does not equal 1 g during quiet periods while the de-biased acceleration equals exactly 1 g.

SA.4. Saturation correction

Our IMU contained two tri-axial accelerometers with different ranges. The primary accelerometer had a range of ± 16 g while the secondary accelerometer had a range of ± 100 g. Although ± 16 g is a large enough range to capture the majority of accelerations at the tibia, iliac crest, and sacrum during running, we wanted to ensure that saturation did not occur, particularly at the tibia [106]. Thus, we used a threshold of $|a| > 15.5$ g and replaced any value above this threshold in our primary accelerometer with the corresponding frame from our secondary accelerometer (these values were highly correlated across the ± 16 g range that they could both measure). The remaining secondary accelerometer data were then discarded.

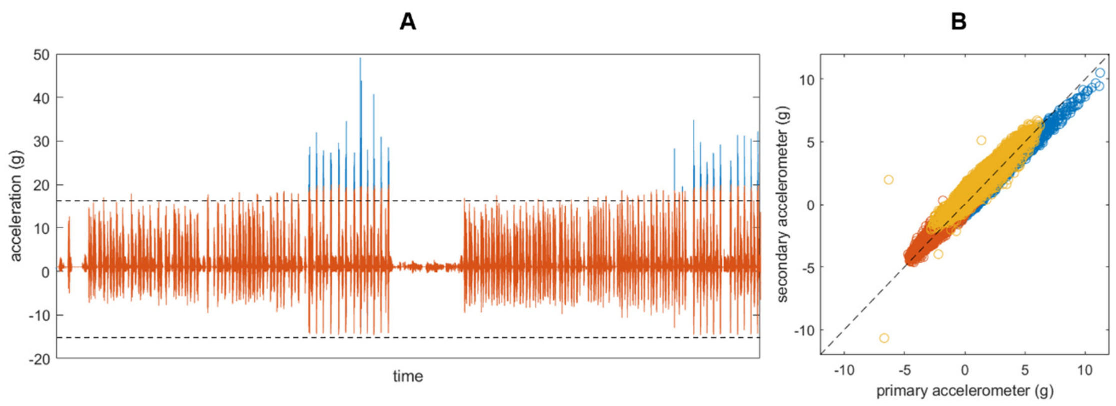

Figure S4. (A) Tibia ~longitudinal axis accelerations from the primary accelerometer (orange) and secondary accelerometer (blue) across the entire data collection for the example participant. The horizontal black line indicates data outside the primary accelerometer's range (defined as |a| > 15.5 g). **(B)** Secondary accelerometer measurements plotted against the primary accelerometer measurements for each axis (different colors). Black dashed diagonal line indicates perfect agreement. In this randomly selected example data, correlations between each axis ranged from r = 0.82 to 0.96. In general, correlations across the ranges shared between primary and secondary accelerometers were ≥ 0.90.

SA.5. Low-pass filtering

Next, accelerations and angular velocities were filtered with a 4th-order 50-Hz low-pass Butterworth filter.

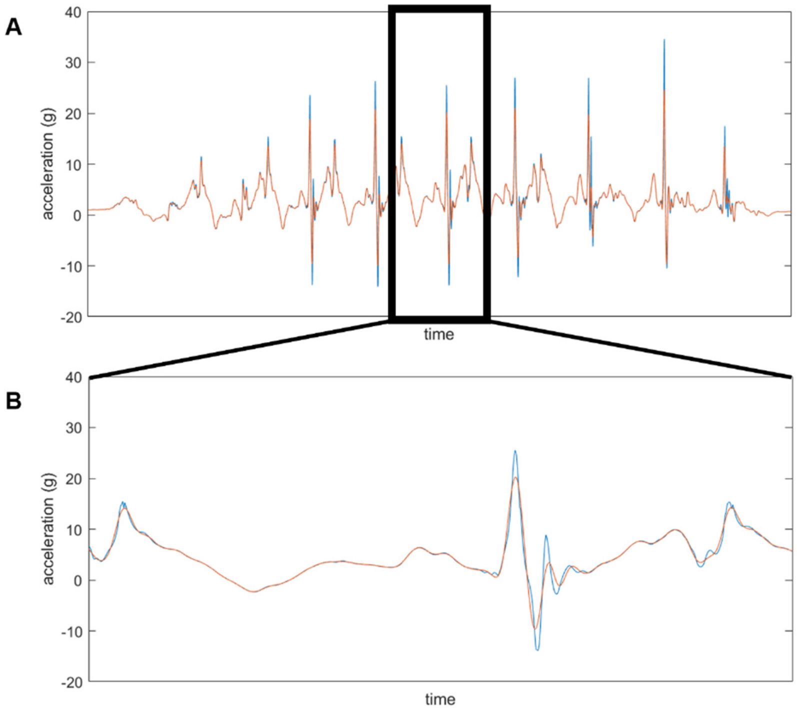

Figure S5: (A) Worst-case example of unfiltered (blue) and filtered (orange) ~longitudinal tibial acceleration (that experienced saturation and is now composed of data from the primary and secondary accelerometers). **(B)** Zoomed in on ~one step to better visualize the differences between the filtered and unfiltered signal. Filter parameters were chosen to qualitatively balance the preservation of major signal features (particularly peak magnitudes and locations) with the removal of high-frequency noise.

SA.6. Drift correction

Angular velocity measured by IMUs is prone to drift. This drift makes it difficult to integrate angular velocities and calculate the orientation of an IMU in space. Several sensor fusion algorithms have been developed to correct this drift including Kalman filters [107], Mahoney filters [108], and Madgwick filters [85]. We explored the use of each of these filters and found that converting our data to quaternion representation and entering it into a Madgwick filter (with beta set to 0.05 and no magnetometer fusion due to the amount of magnetic interference in our lab) was the most successful in eliminating drift in a “worst case” recreation of our experimental conditions (an 80 minute data capture with extreme angular rotations and accelerations and no quiet period corrections yielded a 1.66 rad rotation error accumulated across the entire 80-minute duration). The code we adapted to execute the Madgwick filter is freely available from x-io at:

https://x-io.co.uk/open-source-imu-and-ahrs-algorithms/

and from MATLAB at:

https://www.mathworks.com/products/sensor-fusion-and-tracking.html

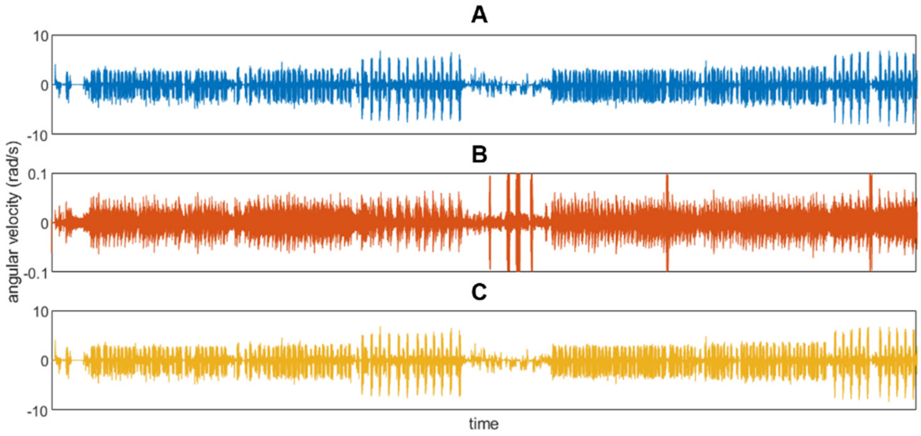

Figure S6: **(A)** Uncorrected angular velocity about the ~longitudinal axis of the sacrum (blue) for the entire data collection of an example participant. **(B)** Difference between the uncorrected and Madgwick filter-estimated angular velocities (orange). **(C)** Madgwick filter-estimated angular velocity about the ~longitudinal axis of the sacrum (yellow).

SA.7. Orientation estimation and gravity subtraction

After drift-correcting the angular velocity with the Madgwick filter, we created a rotation matrix based on the loading of gravity during quiet periods (see A.3 above), then used it to create a “tilt-corrected” coordinate system (see A.8 below). Then, between each quiet period, we used angular velocity to calculate the changes in orientation based on Equations (2) and (3) in McGinnis and Perkins [88]. This provided a rotation matrix from the wearable coordinate system to the “tilt-corrected” coordinate system for each time step.

Using these time-varying rotation matrices, the acceleration data for each frame were expressed in the “tilt-corrected” coordinate system, then 1 g was subtracted from the y-axis (in line with gravity). This procedure removed the gravity component from the accelerometer data. To create the wearable and segment coordinate systems (in the next step), the data were then re-expressed in their original coordinate system using the inverse of the time-varying rotation matrices.

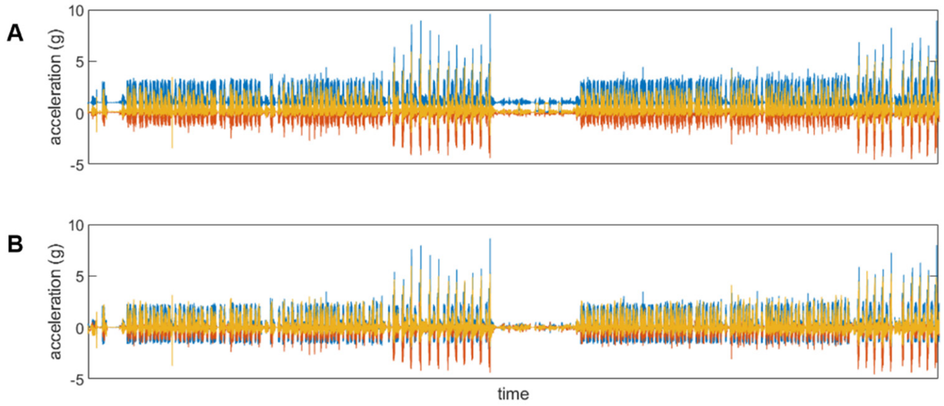

Figure S7. **(A)** Uncorrected acceleration of the sacrum for the entire data collection of a randomly selected example participant (colors represent different axes). **(B)** Acceleration of the sacrum after subtracting 1 g from the y-axis in the "tilt-corrected" coordinate system and then re-expressing in the wearable coordinate system (colors represent different axes).

SA.8. Coordinate system definition

Finally, data were expressed in three different coordinate systems for analysis. First, data were expressed in the Wearable Coordinate System (WCS). This is not the raw coordinate system of the IMU. Rather, all data were corrected with the calibration matrices described in Section A.1 above. These calibration matrices ensured that the data were expressed in orthogonal axes aligned with the IMU housing. The IMU housing was positioned so that during quiet standing, the WCS axes were oriented roughly in the direction of progression (+x), the longitudinal axis (+y), and to the right (+z).

Data were also expressed in a Segment Coordinate System (SCS). This coordinate system was defined using an approach described in the Supplementary Materials of Cain et al. [89], which can be found at:

http://dx.doi.org/10.1016/j.gaitpost.2015.10. 022

In brief, accelerations during a quiet standing trial were used to define a gravity vector (similar to A.3 above), assuming that the segment was aligned with gravity during the standing trial. This gravity-based vector was defined as the proximal–distal axis (+y proximal). Then, a period of steady-state running was manually selected from the dataset. Angular velocities from this period were entered into a principal component analysis and the principal component accounting for the most variability in angular velocity was selected to represent the average axis of rotation. During running, the average axis of rotation is assumed to correspond to the medial–lateral axis. We defined this as the z axis (+z right). The anterior–posterior axis is then defined as the cross-product of y and z (+x anterior). Finally, the z axis is recalculated as the cross-product of x and y to ensure orthogonality. These three unit vectors were then used to create a rotation matrix that transforms data from the WCS to the SCS. The rotation matrix was then plotted to visualize the differences between the WCS and SCS and ensure a solution consistent with our knowledge of the IMU placement had been reached (i.e., the axes were oriented correctly).

Finally, data were expressed in a pseudo-global system similar to Cain et al.'s "tilt-corrected" coordinate system (TCCS) [89]. First, we created a rotation matrix based on the loading of gravity during quiet periods (see Section A.3 above). This rotation matrix expresses data with the y-axis aligned with gravity during quiet standing (+y vertical). Next, the acceleration of each axis was double integrated to obtain displacement, then entered into a principal component analysis. The principal

component accounting for the most variation in displacement was taken as the projection of the direction of progression onto the horizontal plane (+x direction of progression). Then, the projection of the medial–lateral axis onto the horizontal plane was defined as the cross product of x and z (+z right). The x-axis was then recalculated to ensure orthogonality. These three unit vectors were then used to create a rotation matrix and multiplied by the time-varying rotation matrices described in Section A.7 to express the data in the TCCS. Thus, the TCCS data are always expressed with y aligned with gravity, but with x and z free to rotate about y as the participant moves.

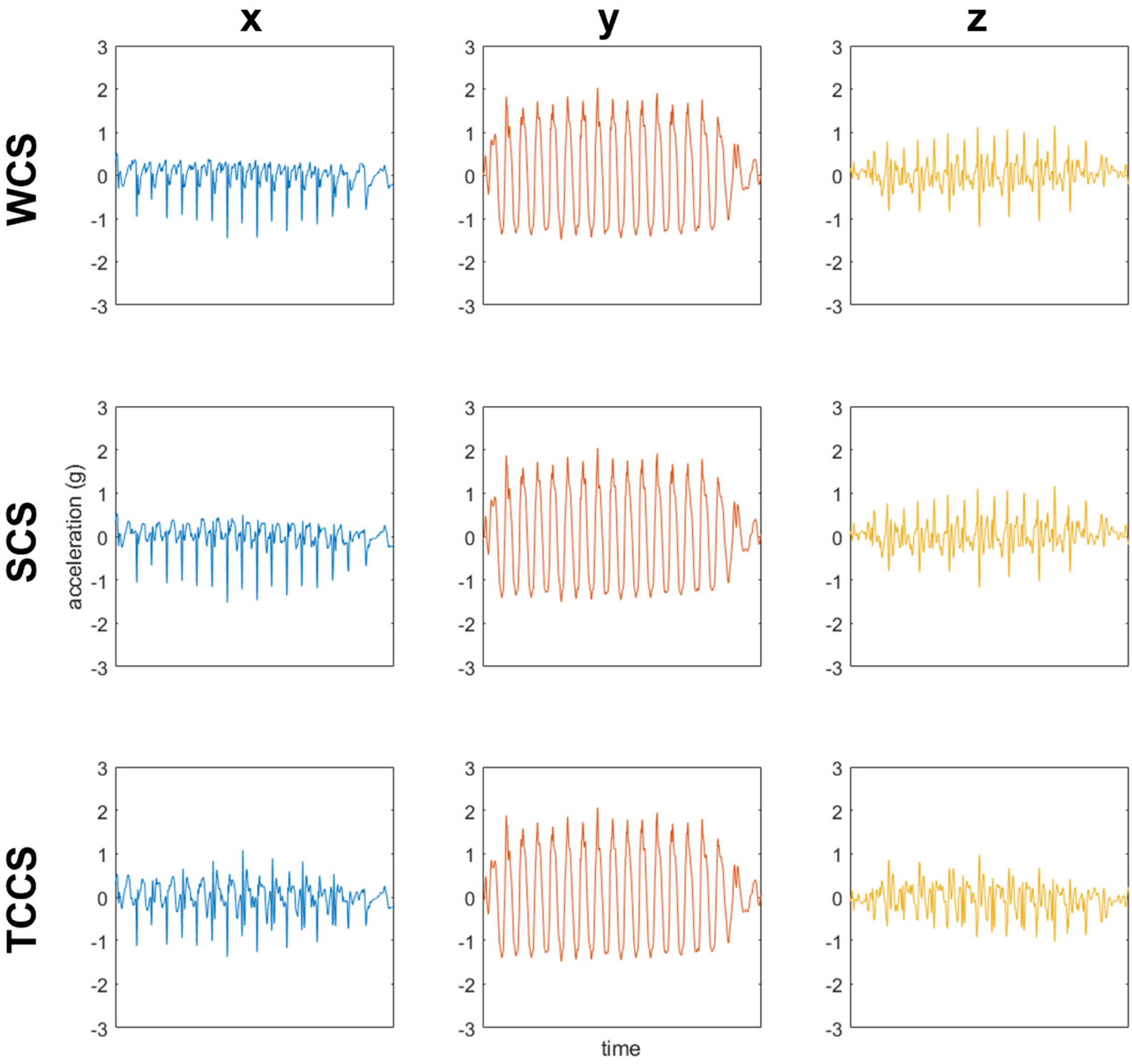

Figure S8: All plots show the sacral accelerations from a randomly selected participant. The top row shows data in the Wearable Coordinate System (WCS). The middle row shows data in the Segment Coordinate System. The bottom row shows data in the Tilt-Corrected Coordinate System (TCCS). The first column shows the x-axis data (blue). The second column shows the y-axis data (orange). The third column shows the z-axis data (yellow). Due to the similarities between all three coordinate systems at the sacrum, the discrepancies are minor.

SB. Description of force estimation methods and their implementation

Here, we provide details on each of the force estimation methods included in our study. Brief descriptions, equations, and figures have been included to show the force estimation process for each method. For additional detail, please refer to the original publications.

SB.1. Neugebauer [73,74]

SB.2. Charry [75]

SB.3. Wundersitz [54]

SB.4. Meyer [76]

SB.5. Gurchiek [55]

SB.6. Thiel [48]

SB.7. Kiernan [77]

SB.8. Kim [78]

SB.9. Pogson [79]

SB.10. Pogson xynorm

SB.11. Day [80]

SB.12. Higgins [40]

SB.13. Veras [63]

All data for the figures in this section were taken from the same randomly selected trial (a 75.3 kg male running 3.20 m/s on the 'floor' surface with a rear foot strike angle of 0.44 rad). For clarity, the different coordinate systems used across the original publications were standardized to the conventions described in our main paper and Supplementary Materials A. All example data are shown in the SCS regardless of the original coordinate system used by the method, but Supplementary Materials C demonstrates the effects of different coordinate systems on the data.

Finally, our implementation of each of these methods is freely available at:

https://github.com/DovinKiernan/MTFBWY_running_vGRF_from_a

To use these implementations, simply feed the acceleration data during a single stance into the provided function as an f × 4 matrix where f is the frame and the columns correspond to time (in ms) and accelerations in the x, y, and z axes (in g).

SB.1. Neugebauer method

Neugebauer et al. published two papers using linear regression to estimate the maximum vertical GRF from accelerometers worn on the hips of children and adults [73,74]. To do so, they used the generalized (non-participant-specific) equation:

$$\ln(F_{y,max}) = \alpha_0 + \alpha_1 a_{WCS,y,max} + \alpha_2 m + \alpha_3 L + \alpha_4 L a_{WCS,y,max} \quad \text{Equation S9}$$

where m corresponds to participant mass and L corresponds to the type of locomotion (with walking equal to 0 and running equal to 1). Thus, for running, their equation simplifies to:

$$\ln(F_{y,max}) = (\alpha_0 + \alpha_3) + (\alpha_1 + \alpha_4)a_{WCS,y,max} + \alpha_2 m$$ Equation S10

$$\ln(F_{y,max}) = c_1 + c_2 a_{WCS,y,max} + c_3 m$$ Equation S11

$$F_{y,max} = e^{c_1 + c_2 a_{WCS,y,max} + c_3 m}$$ Equation S12

where c_1, c_2, and c_3 are constants replacing $(\alpha_0 + \alpha_3)$, $(\alpha_1 + \alpha_4)$, and α_2, respectively. Note that Neugebauer and colleagues published a third paper estimating maximum vertical GRF from the accelerations of soldiers carrying a load during walking that can also be reduced to Equation (S12) if carrying a load of 0 [109].

Here, we made two assumptions: (1) that Neugebauer defined their WCS with ~vertical as positive, and (2) that $F_{y,max}$ is analogous to $F_{y,second}$. We then used a leave-one-out cross-validation to iteratively calculate the error for one participant using the c_1, c_2, and c_3 values calculated from the other 73 participants while the final c_1, c_2, and c_3 values were calculated using the data from all 74 of our participants. Also note that in their 2012 and 2014 papers, Neugebauer and colleagues were unable to synchronize their accelerometers and force plates and thus used $a_{WCS,y,max}$ averaged across 30 s and 10 s, respectively, in those papers. In contrast, we used $a_{WCS,y,max}$ during the single stance for which $F_{y,second}$ is being estimated.

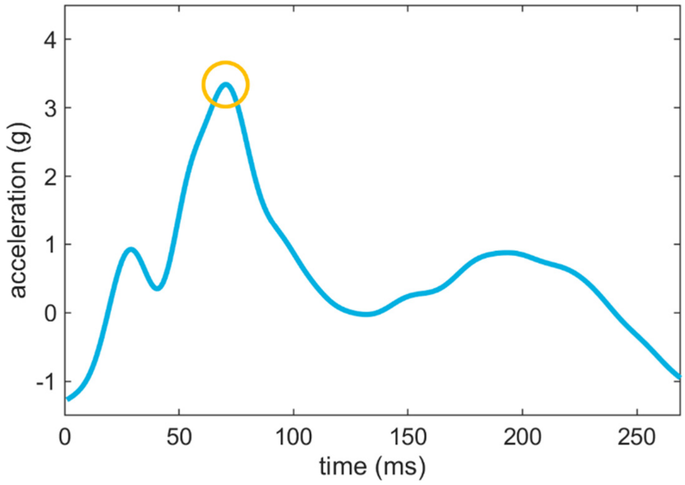

Figure S9: Hip y-axis acceleration (proximal–distal in the SCS; ~ longitudinal in the WCS; blue line) maxima (yellow circle) and participant mass are entered into Equation (S12) to estimate the vertical GRF second peak magnitude.

SB.2. Charry method

Charry et al. [75] placed a ± 24 g accelerometer on the medial midshaft of three participants tibiae and measured $a_{WCS,y}$ at 100 Hz while the participants ran 1.7 to 7.2 m/s overground. Charry et al. evaluated the efficacy of four potential acceleration variables to predict the vertical GRF second peak magnitudes: (1) heel-strike, (2) initial peak acceleration, (3) minimum peak (specified as the maximum in their paper but the minimum here based on differences in coordinate conventions), and (4) peak-to-peak (Figure S10A). They found that minimum peak acceleration was the best predictor of vertical GRF second peak magnitude and thus discarded the other three predictor variables. Charry et al. evaluated both a linear and logarithmic prediction equation and found that the logarithmic equation better predicted vertical GRF second peak magnitude; thus, they discarded the linear fit and used:

$$F_{y,second} = log_2(-a_{WCS,y,min} + 1) \quad \text{Equation S13}$$

where 1 was added to acceleration values to prevent taking the log of a negative value. Charry et al. then added additional terms to their prediction equation to create slopes and intercepts that were a function of participant mass, using the form:

$$F_{y,second} = s(m) * log_2(-a_{WCS,y,min} + 1) + i(m) \quad \text{Equation S14}$$

where s(m) and i(m) were found by taking the participant- and leg-specific slopes and intercepts (respectively) from Equation (S13) (Figure S10B) and plotting them against participant mass (Figure S10C), then defining s(m) and i(m) as the equation for that line. Thus, Equation (2) can be expanded to the form:

$$F_{y,second} = (s_1 m + s_2) * log_2(-a_{WCS,y,min} + 1) + (i_1 m + i_2) \quad \text{Equation S15}$$

The original s(m) and i(m) were found on the three participants in Charry et al.'s study. Here, we used a leave-one-out cross-validation to iteratively calculate the error for one participant using s(m) and i(m) terms calculated from the other 73 participants while the final s(m) and i(m) terms were calculated using the data from all 74 of our participants.

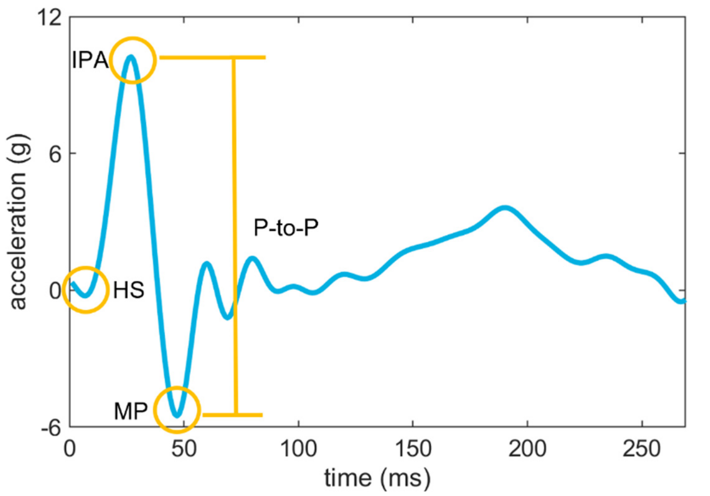

Figure S10: (A) Tibia y-axis acceleration (proximal–distal in the SCS; ~longitudinal in the WCS; blue line) features Charry et al. used to predict vertical GRF second peak magnitude. HS = heel strike, IPA = initial peak acceleration, MP = minimum peak (originally specified as a maximum but here as a minimum due to differences in coordinate conventions), and P-to-P = peak-to-peak. 'Minimum peak' was found to be the best predictor and all other variables were discarded.

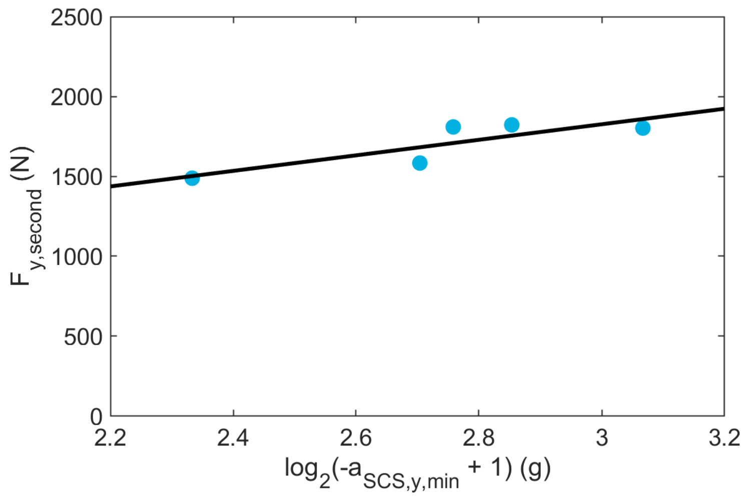

(B) Fit for Charry et al.'s Equation (1) for a single leg from the selected participant. Each blue point represents one stance. To account for differences in coordinate conventions, the negative value of the minimum peak was used. Although 1 was added to all accelerations, 0.2% of results when using the SCS were still imaginary numbers. These results were discarded from the analysis.

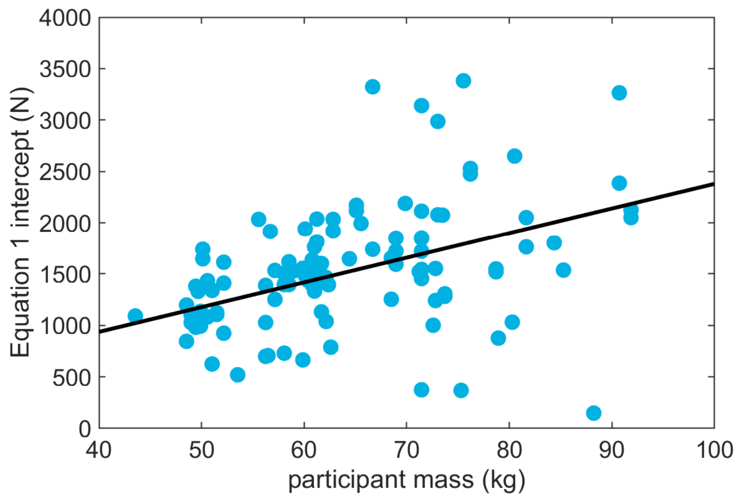

(C) Participant- and leg-specific intercepts from the line of best fit in the preceding figure plotted against participant mass for each participant and leg (blue points). The equation describing the line of best fit for the black line was used to define the i(m) term in Charry et al.'s Equations (S14) and (S15). A similar procedure was conducted using the slopes from the previous step (Figure S9B) to define the s(m) term.

SB.3. Wundersitz method

Wundersitz et al. [54] measured the vertical GRF and second thoracic vertebra acceleration from 17 competitive team sport athletes while they ran either in a straight line or with a change in direction. To estimate force, they multiplied acceleration by mass. They then filtered the estimated force with a 4th-order low-pass Butterworth filter of either 10, 15, 20, or 25 Hz and extracted the peak value. We assumed that the peak force they were estimating corresponded to the vertical GRF second peak such that:

$$F_{y,second} = 9.8ma_{WCS,y,max} \quad \text{Equation S16}$$

where m is mass in kg and $a_{WCS,y,t}$ is acceleration in g (with 1 g = 9.8 m/s^2).

Here, we adapted this upper back approach to our sacrum data.

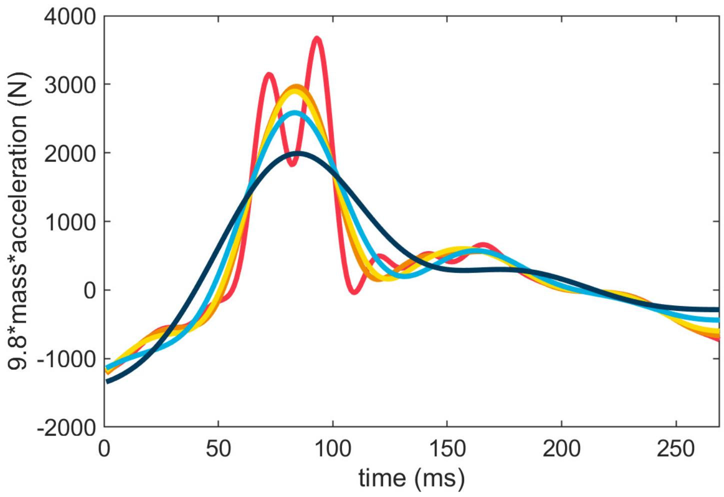

Figure S11: Sacrum y-axis acceleration (proximal–distal in the SCS; ~longitudinal in the WCS) was multiplied by participant mass then low-pass filtered at 10, 15, 20, 25-Hz, and no filter (dark blue, light blue, yellow, orange, and red lines, respectively). Maxima were then identified and used to estimate the vertical GRF second peak.

SB.4. Meyer method

Meyer et al. [76] had 13 moderately active children perform a range of tasks including jogging and running while they wore triaxial accelerometers on their right hip. Meyer et al. did not explicitly define their coordinate convention but reported extracting the “minimum acceleration of the vertical axis”. We assumed that this corresponded to a maximum in the ~longitudinal axis as defined in our WCS (see Figure S9 above). Acceleration and force signals were not time synchronized; thus, Meyer et al. extracted and averaged their acceleration minima across 8–15 steps per trial. The correlation between these average accelerations and the “peak impact forces in the vertical plane” were then explored (which we interpreted as the maximum vertical GRF and *not* the vertical GRF first peak magnitude based on their reported magnitudes and on the fact that their other tasks would not have an “impact peak”, as the term is commonly used in the running literature). Meyer et al. also explored sex, age, weight, height, and leg length as potential explanatory variables but found that they were not significant predictors. Thus, they ultimately used a correlation between the mean $a_{WCS,y,max}$ across 8–15 steps and the $F_{y,max}$ of a single stance on the force plate (expressed in body weights), observing an r^2 of 0.81. This can be expressed as:

$$\frac{F_{y,max}}{9.8m} = a_{WCS,y,max} \qquad \text{Equation S17}$$

and rearranged as:

$$F_{y,second} = 9.8ma_{WCS,y,max} \qquad \text{Equation S18}$$

where m is mass in kg and $a_{WCS,y,max}$ is acceleration in g (with 1 g = 9.8 m/s^2), and with the assumption that the $F_{y,max}$ they attempted to estimate corresponded to $F_{y,second}$ at the running speeds they studied. This equation resembles the one used by Wundersitz et al. [54] (above) but with different data entered into the equation.

We used Equation (S18) to estimate $F_{y,second}$ across our 74 participants using the $a_{WCS,y,max}$ from the same stance the force was taken from.

SB.5. Gurchiek method

Gurchiek et al. [55] had 15 participants perform sprint start and change of direction tasks while wearing an IMU on their sacrum. Data were low-pass filtered at 30 Hz then re-expressed in a GCS. The force time series and average across stance were estimated by scaling acceleration by mass:

$$F_{y,t} = 9.8ma_{GCS,y,t} \qquad \text{Equation S19}$$

where m is mass in kg and $a_{GCS,y,t}$ is acceleration in g (with 1 g = 9.8 m/s^2). This equation resembles the one used by Wundersitz et al. [54] and Meyer et al. [76] (above) but with different data entered into the equation.

B.6. Thiel method

Thiel et al. [48] placed IMUs above the medial malleoli of three elite sprinters and had them sprint 50 m on a track with embedded force plates. They calculated the maximum vertical GRF as:

$$F_{y,max} = c_1(n)a_{WCS,x} + c_2(n)a_{WCS,y} + c_3(n)a_{WCS,z} \qquad \text{Equation S20}$$

where c_1, c_2, and c_3 are coefficients that varied linearly as a function of stride number n and where we assumed that $F_{y,max}$ was the vertical GRF second peak and that each "acceleration component" was the maximum value observed during stance, such that:

$$F_{y,second} = (c_{1a}n + c_{1b})a_{WCS,x,max} + (c_{2a}n + c_{2b})a_{WCS,y,max} + (c_{3a}n + c_{3b})a_{WCS,z,max}$$
Equation S21

Thiel et al. noted that this stride-varying approach was suitable until the maintenance (steady-state) phase, where an approximately constant relation between acceleration and force is expected. Thus, during steady state running, the coefficients could be assumed constant, and the equation can be simplified as:

$$F_{y,second} = c_1 a_{WCS,x,max} + c_2 a_{WCS,y,max} + c_3 a_{WCS,z,max}$$
Equation S22

Here, we assumed that (1) participants were running at a steady-state and therefore used the constant expression Equation (S22), and that (2) Thiel et al. used a WCS with ~vertical, ~anterior, and ~medial defined as positive (although they did not fully describe their coordinate convention, they did provide time-series acceleration figures that suggest this was their convention. Note that their text suggests that the right and left medial–lateral axes were defined with positive in opposite directions; however, we inferred that the minima was taken from the right and the maxima from the left, effectively making the method the maxima from the ~medial direction).

To execute this method, we used a leave-one-out cross-validation to iteratively calculate the error for one participant using the c_1, c_2, and c_3 coefficients calculated using the other 73 participants while the final c_1, c_2, and c_3 coefficients were calculated using the data from all 74 of our participants.

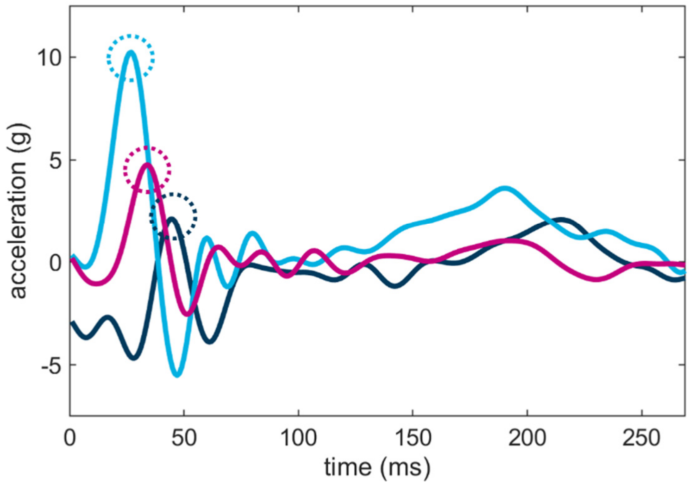

Figure S12: Tibial accelerations from the x- (anterior–posterior in the SCS; ~direction of progression in the WCS; dark blue line), y- (proximal–distal in the SCS; ~longitudinal in the WCS; light blue line), and z-axes (medial–lateral in the SCS; ~right in the WCS; pink line) maxima were found (dashed circles). Note that we inferred that the largest medial value was taken from the z-axis; thus, for right stances (such as the one shown here), the z-axis was multiplied by −1. These values were multiplied by the coefficients c_1, c_2, and c_3 to estimate the vertical GRF second peak magnitudes.

SB.7. Kiernan method

Building on work by Neugebauer et al. [75,74,109], Kiernan et al. [77] had 40 participants run overground at slow, typical, and fast speeds while recording accelerations from their iliac crests and sacra. In an effort to estimate both the first and second peak of the vertical GRF, the $a_{SCS,y}$ signal from each location was divided into signals composed of 0–8 Hz 'LoF' frequency content and a ≥10 Hz

‘HiF’ frequency content [91]. For each of the two sensor locations, maxima from the LoF and HiF signals were found and entered into a linear regression along with the sex, height, mass, and leg length to estimate the log transformed vertical GRF first and second peaks. Kiernan et al. also explored models, including the speed and participant as fixed and random effects. They found that this could improve model performance; however, we used the generalizable form of their model:

$$\ln(F_{y,second}) = c_1 + c_2 a_{SCS,y,LoF,max} + c_3 a_{SCS,y,HiF,max} + c_4 s + c_5 m + c_6 h + c_7 g \quad \text{Equation S23}$$

which can be rearranged as:

$$F_{y,second} = e^{c_1 + c_2 a_{SCS,y,LoF,max} + c_3 a_{SCS,y,HiF,max} + c_4 s + c_5 m + c_6 h + c_7 g} \quad \text{Equation S24}$$

where $c_{1:7}$ are constants, $a_{SCS,y,LoF,max}$ and $a_{SCS,y,HiF,max}$ are low and high frequency acceleration maxima, s is the self-reported participant sex (with female = 0, male = 1, and no non-binary reported), m is participant mass, h is participant height, and g is the height of the greater trochanter.

To replicate this method, we used a leave-one-out cross-validation to iteratively calculate the error for one participant using $c_{1:7}$ values found using the other 73 participants while the final $c_{1:7}$ values were found using data from all 74 of our participants. Note that the original model published by Kiernan et al. was developed with a subset of the current sample (40 of our 74 participants).

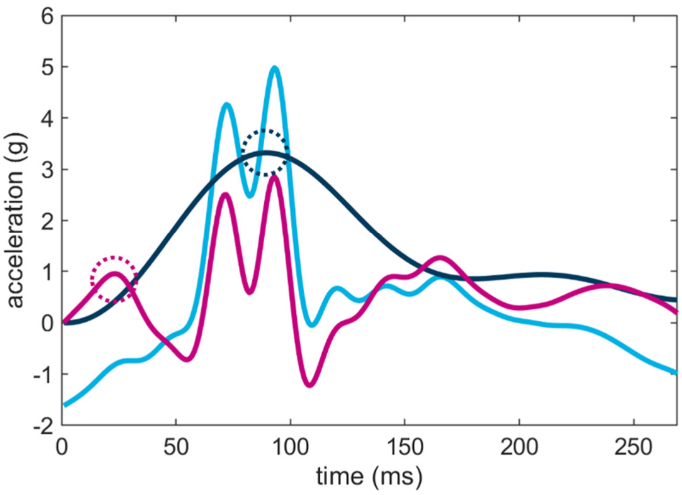

Figure S13: Sacrum y-axis acceleration (proximal–distal in the SCS; ~longitudinal in the WCS; light blue line) and its low frequency (dark blue line) and high frequency components (pink line). The peak in the low frequency was identified (dark blue circle), then the earliest occurring peak in the high frequency between the start of the stance and the low frequency peak was identified (pink circle). These peaks were entered into a linear regression to estimate vertical GRF second peak magnitude.

SB.8. Kim method

To estimate GRF from acceleration during running, Kim et al. [78] used a feed-forward neural network (FFNN). They proposed two models: a “SLIP” model (spring loaded inverse pendulum) that estimated GRF from sacrum displacement (double integrated acceleration) and a “rigid dynamics” model that estimated GRF from sacrum acceleration.

Kim et al. tested these two models on seven participants who ran on a treadmill while sacral acceleration was recorded via motion capture. Triaxial accelerations were low-pass filtered at 10 Hz with a 5th-order Butterworth filter. Accelerations (or displacements) at a single time point, and the time point itself, were then entered into an FFNN with a single 10-node hidden layer to estimate triaxial

forces normalized to body weight. Here, however, we followed the approach laid out in Equation (S16) (above) and instead multiplied the acceleration input by mass to estimate non-normalized force.

Kim et al. did not report a normalization procedure for the acceleration inputs to the FFNN but based on their previous work [34], we assumed that data were normalized by maximum acceleration so that all values ranged from 0 to 1. Similarly, we inferred from their figures that time was expressed as percent stance from 0 to 100. Few details were provided regarding model parameters; thus, we assumed that log-sigmoid and pure linear activation functions were used in the hidden and output layers and that a 2000 epoch Levenberg–Marquardt function was used for training.

To evaluate their model, we used a leave-one-out approach and iteratively trained a network to minimize the mean square errors on 73 participants using an 80–20 training–validation split. We then calculated the errors between the estimated and actual $F_{y,t}$ on the remaining participant. Weights and biases for the final model were calculated by training the FFNN on all 74 participants.

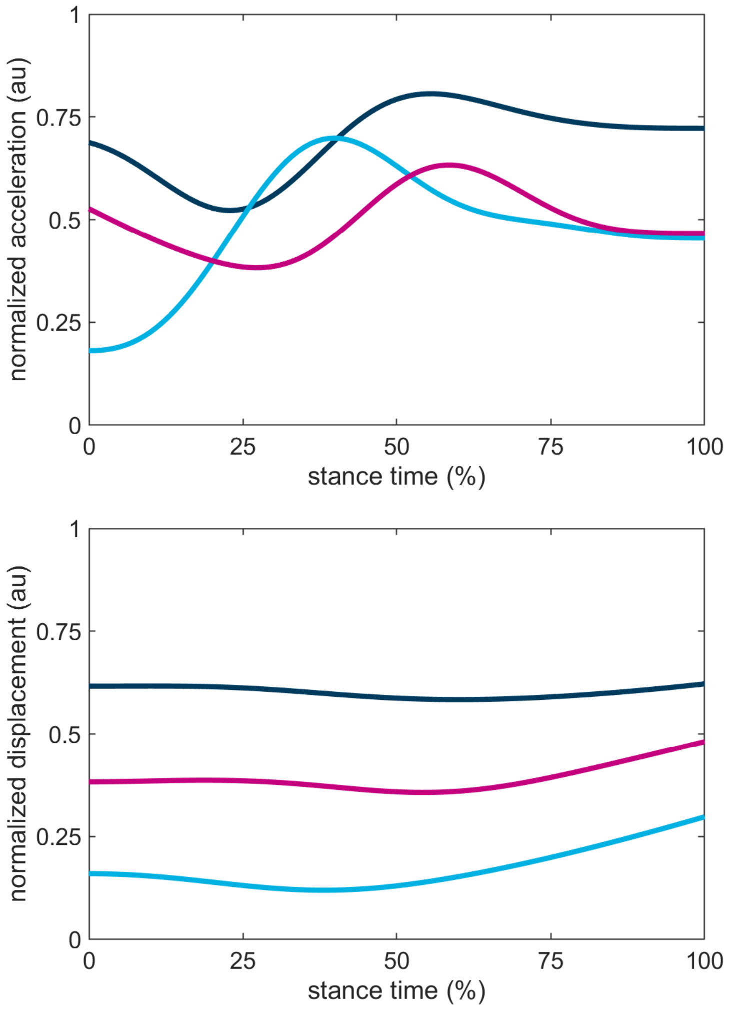

Figure S14: (A) Sacral accelerations normalized from 0 to 1 based off the minimum and maximum observed values in the x- (anterior–posterior in the SCS; ~direction of progression in the WCS; dark blue line), y- (proximal–distal in the SCS; ~longitudinal in the WCS; light blue line), and z-axes (medial–lateral in the SCS; ~right in the WCS; pink line). Tri-axial values from each time point were used to estimate corresponding vertical GRF values.

(B) Sacral displacements normalized from 0 to 1 based off the minimum and maximum observed values in the x- (anterior-posterior in the SCS; ~direction of progression in the WCS; dark blue line), y- (proximal-distal in the SCS; ~longitudinal in the WCS; light blue line), and z-axes (medial-lateral in the SCS; ~right in the WCS; pink line). Tri-axial values from each time point were used to estimate the corresponding vertical GRF values.

SB.9. Pogson method

Pogson et al. [79] measured resultant forces and upper back accelerations while 15 team sport athletes ran overground. Given our goal of estimating vertical GRFs, we instead used the y-axis force and acceleration. Accelerations were segmented by stance, then zero-padded to the duration of the longest stance (we zero-padded to 0.4 s to ensure that the method could accommodate all future data). Acceleration and force data were then entered into a principal component analysis. The

acceleration principal components (PCs) and signal duration were then entered into a multilayer perceptron (MLP) that was trained to estimate the force PCs. Pogson et al. used a stochastic particle swarm optimization to determine the number of inputs (acceleration PCs), hidden layers, number of nodes in each layer, and outputs (force PCs) that minimized the force PC estimation error. They reported that all optimizations returned similar values but found slightly better results with an MLP using six acceleration PCs, with five hidden layers containing 45, 36, 45, 82, and 40 nodes, and trained to estimate eight force PCs. The force PCs were then used to reconstruct the force signal. We assumed that the input data were normalized from 0 to 1 and that the sigmoid activation functions were used between each of the layers, except for the output layer, where a linear activation function was used.

The reconstructed force signal could vary in duration from the input and target signals. To address this issue, Pogson et al. reported appending the signal duration to the output layer (as a quantity to be estimated), then trimming the force signal to the estimated duration. During development, however, we observed that this approach often led to input and output signals of different durations, increasing error (consistent with Pogson et al.'s Figure 4). Given that the estimated force should always have the same duration as the acceleration input (because both are segmented based on the stance onset and offset), we trimmed any values following the point where the estimated force fell below 10 N, then interpolated the force signal to match the duration of the input signal. This approach greatly reduced the errors.

To evaluate Pogson's method, we tuned the hyperparameters of the MLP solver using a grid search, then used a leave-one-out approach to iteratively train a network that minimized mean square errors on 73 participants using an 80–20 training–validation split. We then calculated the errors between estimated and actual $F_{y,t}$ on the remaining participant. Weights and biases for the final model were calculated by training the MLP on all 74 participants.

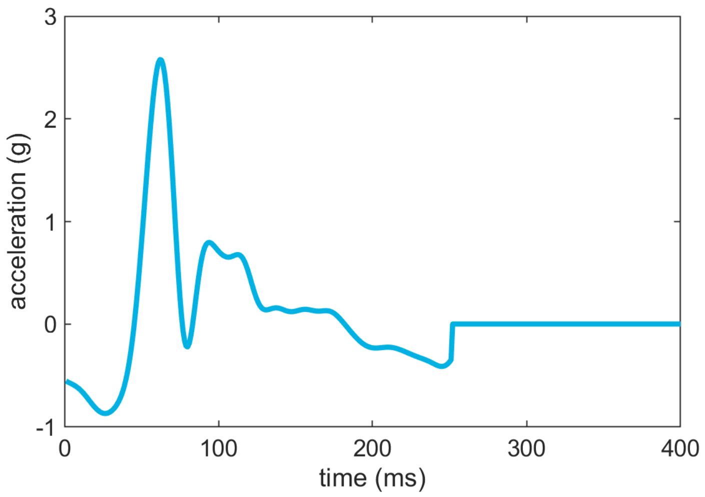

Figure S15: (A) Sacral acceleration in the y-axis (proximal–distal in the SCS; ~longitudinal in the WCS; light blue line) zero-padded to 400 ms.

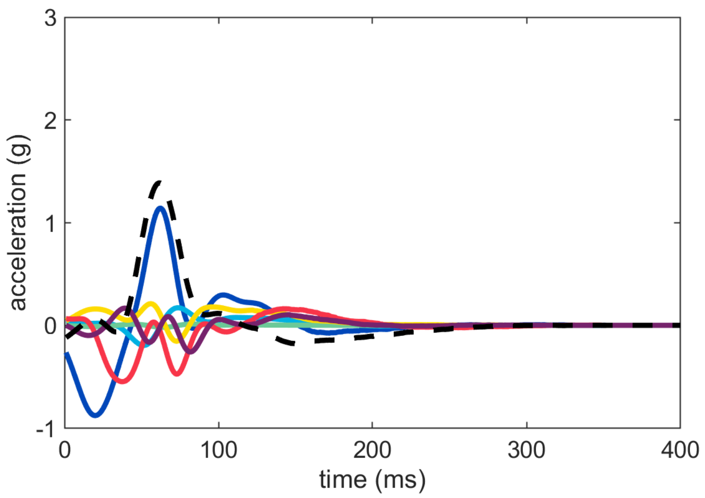

(B) The first six principal component reconstructions for the example trial (solid lines; PC scores multiplied by coefficients) and mu (dashed line; the estimated means of each time point across the sample). The sum of these seven lines reconstructs the original signal. The scores used to calculate the solid lines were entered into the machine learning model to estimate the vertical GRF PC scores.

SB.10. Pogson xynorm method

We observed that the Pogson et al. [79] method was promising but that the majority of errors originated from differences between the duration of the original force signal and the estimated force signal. To deal with this source of error, we modified Pogson et al.'s approach: instead of zero-padding the data, we standardized all signals to 101 time points and followed the approach laid out in Equation (S16) (above), multiplying the acceleration input by mass before applying the principal component analysis. After estimating the PC scores and reconstructing the signal, it was rescaled to its original duration.

To evaluate this model, we tuned the hyperparameters of the MLP solver using a grid search, then used a leave-one-out approach to iteratively train a network that minimized the mean square errors on 73 participants using an 80–20 training–validation split. We then calculated errors between the estimated and actual $F_{y,t}$ on the remaining participant. Weights and biases for the final model were calculated by training the MLP on all 74 participants.

SB.11. Day method

Day et al. [80] had 30 NCAA Division 1 cross country runners run on an instrumented treadmill while wearing an IMU clipped to their posterior waistband. They filtered $a_{WCS,y}$ at 5, 10, and 30 Hz. Other filter parameters were not reported; thus, we assumed that they used the same 8th-order low-pass Butterworth filter that was used to filter their force data. Then, using an approach similar to Wundersitz et al. [54], Meyer et al. [76], and Gurchiek et al. [55], they multiplied acceleration by mass to estimate the force:

$$F_{y,t} = 9.8ma_{WCS,y,t} \quad \text{Equation S25}$$

where m is mass in kg and $a_{WCS,y,t}$ is acceleration in g (with 1 g = 9.8 m/s^2) at time t. This equation resembles the one used by Wundersitz et al. [54], Meyer et al. [76], and Gurchiek et al. [55] (above) but with different data entered into the equation (namely, the data had different filtering, a different coordinate system, and came from a different sensor location).

For this method, we found that the aggressive filtering (particularly the 5 Hz) led to signal distortion when using stance-segmented data and thus, in contrast to the other methods, here, we

filtered *before* segmenting by stance. If using this method, please be aware that your data must be filtered before passing your stance-segmented data into our provided code.

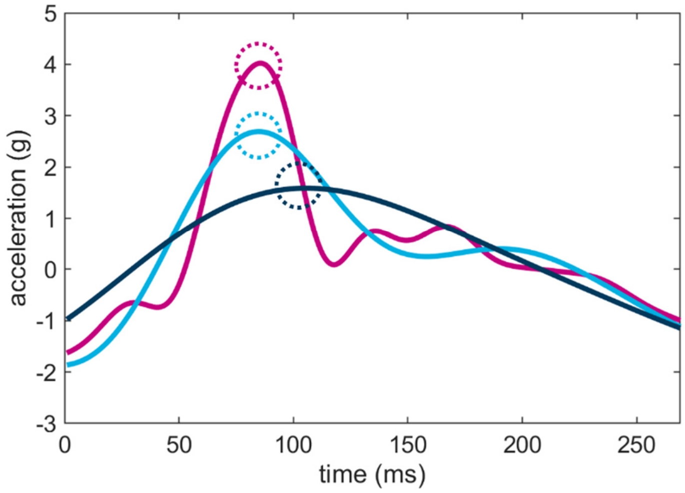

Figure S16: Sacrum y-axis acceleration (proximal–distal in the SCS; ~longitudinal in the WCS) low-pass filtered at 5, 10, and 30-Hz (dark blue, light blue, and pink lines, respectively) maxima (dashed circles) were multiplied by participant mass to estimate the vertical GRF second peak magnitude.

SB.12. Higgins method

Higgins et al. [40] had 30 participants perform a range of tasks including jogging and running down a 23 m pathway with an embedded force plate while wearing an accelerometer on their right hip and ankle. Higgins et al. took the maximum acceleration in $a_{WCS,y}$ during each stance and entered it into linear mixed models (note that their coordinate convention was not explicitly defined outside of stating that the vertical vector is "typically in line with the majority of gravity related loading"; based on this statement, we inferred that they used a WCS with the ~vertical direction defined as positive). In addition to $a_{WCS,y,max}$, Higgins et al. explored several variables including sex, age, and "activity code" as potential predictors of either average vertical loading rate or vertical GRF first peak magnitude. Potential predictors that were not significant were iteratively removed, leading to the development of the following equations:

$$F_{y,first} = \alpha + \beta_1 a_{WCS,y,max} + \beta_2 A \quad \text{Equation S26}$$

$$\frac{dy}{dx} F_{y,first} = \alpha + \beta_1 a_{WCS,y,max} + \beta_2 A + \beta_3 y \quad \text{Equation S27}$$

where A is activity code (jogging = 1, running = 2) and y is age in years. Here, we assumed that a single type of activity (running) resulted in a constant activity code A. Furthermore, although Higgins et al. originally specified α as a participant-specific intercept, our goal here was to produce a generalizable method, thus we simplified their approach to:

$$F_{y,first} = c_1 + c_2 a_{WCS,y,max} \quad \text{Equation S28}$$

$$\frac{dy}{dx} F_{y,first} = c_1 + c_2 a_{WCS,y,max} + c_3 y \quad \text{Equation S29}$$

where c_1 corresponds to $(\alpha + \beta_2 A)$ with A being constant and where c_2 and c_3 correspond to β_1 and β_3, respectively.

Note that Higgins et al. also attempted to estimate the average vertical loading rate and vertical GRF first peak magnitude using the same approach but with shank accelerations. They found that hip accelerations provided better estimates across the activities they studied and thus did not report the full details on their shank models (despite reporting that $a_{WCS,y,max}$ measured at the shank had the highest observed correlation with vertical loading rate during running). Thus, to replicate their shank method, we assumed that it was identical to their hip method.

We used a leave-one-out cross-validation to iteratively calculate the error for one participant using the c_1, c_2, and c_3 values found using the other 73 participants while the final c_1, c_2, and c_3 values were found using data from all 74 of our participants.

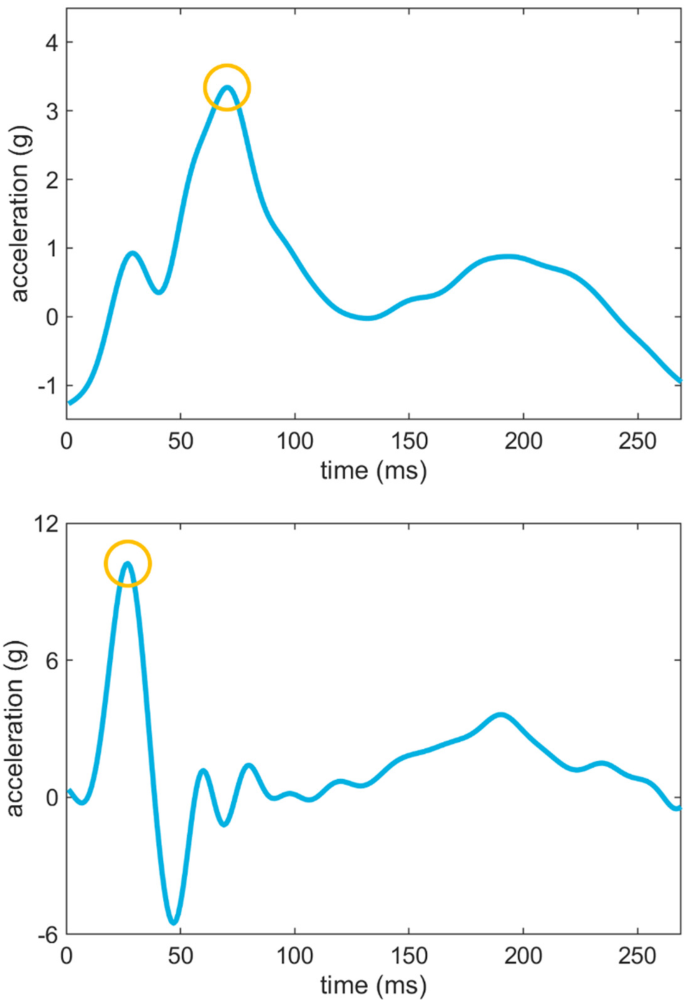

Figure S17: (A) Hip y-axis acceleration (proximal–distal in the SCS; ~longitudinal in the WCS; blue line) maxima (yellow circle).

(B) Shank y-axis acceleration (proximal–distal in the SCS; ~longitudinal in the WCS; blue line) maxima (yellow circle).

SB.13. Veras method

Veras et al. [63] had 131 participants walk and run on an instrumented treadmill while wearing accelerometers on their tibiae, hips, and sacra. Peak accelerations and jerks (first derivative of acceleration) were extracted from $a_{WCS,y}$ and $a_{WCS,res}$ signals low-pass filtered with a 4th-order 20-Hz Butterworth filter. These values were entered into linear regressions along with participant mass to predict $F_{y,max}$ and $\frac{dy}{dx}F_{y,first}$. Body mass index and body mass index "category" were also explored as potential predictor variables, but did not improve model performance. On the other hand, models

were improved by the inclusion of random effects for speed and participant. To maintain generalizability, however, we omitted these variables, resulting in the equations:

$$F_{y,max} = c_1 + c_2 a_{WCS,y,max} + c_3 m + c_4 m a_{WCS,y,max} \quad \text{Equation S30}$$

$$\frac{dy}{dx} F_{y,first} = c_1 + c_2 \frac{dy}{dx} a_{WCS,y,max} + c_3 m + c_4 m \frac{dy}{dx} a_{WCS,y,max} \quad \text{Equation S31}$$

where c_1, c_2, c_3, and c_4 are constants and m corresponds to participant mass.

Here, we assumed that $F_{y,max}$ is analogous to $F_{y,second}$. We then used a leave-one-out cross-validation to iteratively calculate error for one participant using the c_1, c_2, c_3, and c_4 values found using the other 73 participants while the final c_1, c_2, c_3, and c_4 values were found using data from all 74 of our participants.

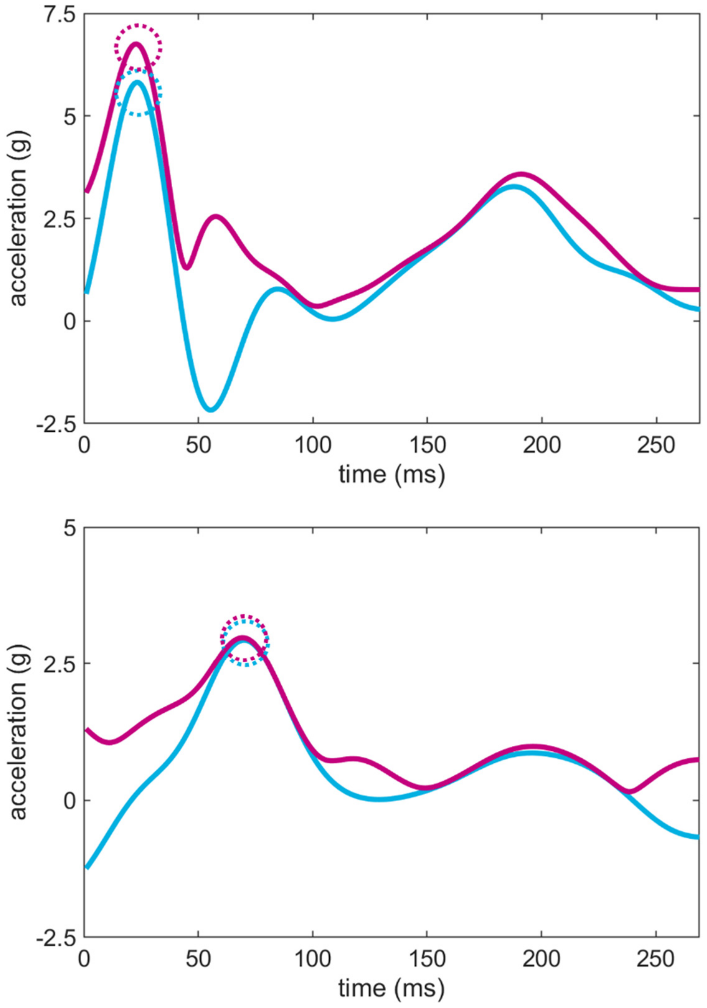

Figure S18: (A) Shank y-axis (proximal–distal in the SCS; ~longitudinal in the WCS; blue line) and resultant (pink line) accelerations and maxima (dashed circles).

(B) Hip y-axis acceleration (proximal–distal in the SCS; ~longitudinal in the WCS; blue line) maxima (yellow circle).

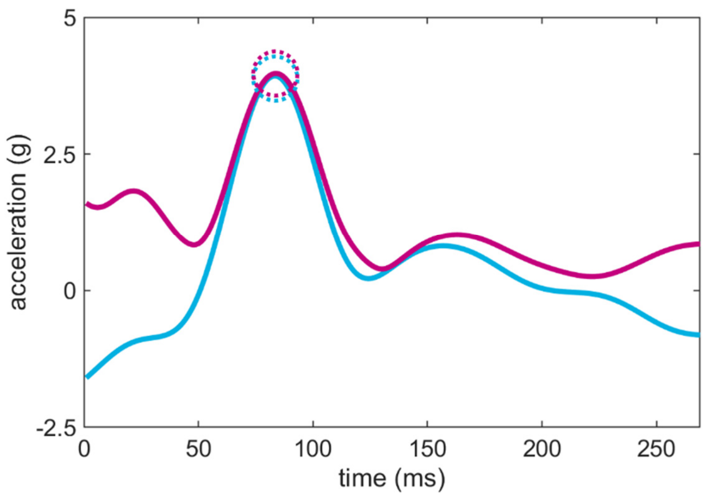

(C) Sacrum y-axis acceleration (proximal–distal in the SCS; ~longitudinal in the WCS; blue line) maxima (yellow circle).

SC. Results across wearable, segment, and tilt-corrected coordinate systems

Despite the fact that the majority of the publications described in Section B expressed their acceleration data in the WCS (11 of 13), we presented our results in the main paper based on acceleration data expressed in the SCS. We made this choice based on an a priori expectation that the WCS would be more prone to errors due to IMU misplacement/misalignment, differences in participant morphology, and/or differences in participant posture/kinematics. We did, however, also execute the same analyses presented in the main paper, but with WCS and TCCS acceleration data as inputs.

Overall, we found that, for all linear regression and machine learning methods that had coefficients, weights, or biases fit to the specific input data (i.e., had different values for WCS, SCS, and TCCS), there were no systematic effects. In contrast, for methods that simply multiplied acceleration by mass, WCS error was systematically higher. This result was consistent across all of the estimated features (first peak, loading rate, second peak, average, and time series); thus, we only present our time series results here for the purposes of illustration (Figure S19).

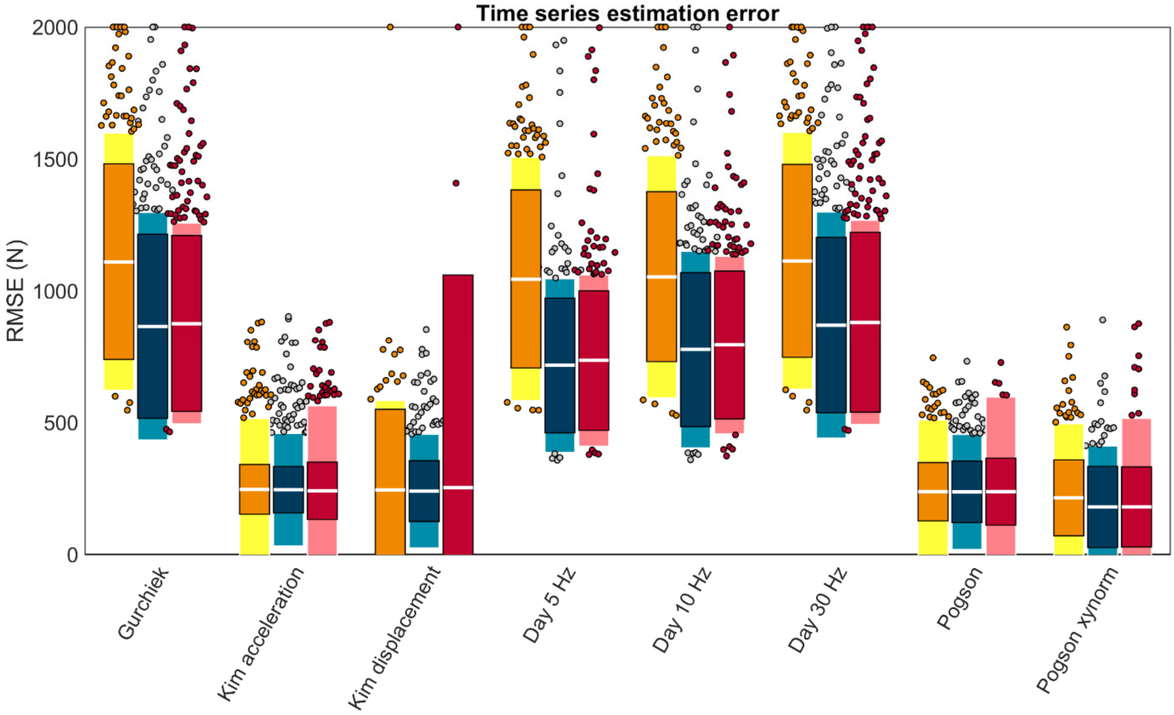

Figure S19: Mean RMSE for each method capable of estimating vertical GRF time series based on acceleration inputs. Orange and yellow represent estimations made with models trained on WCS input data, blue on SCS input data (identical to the main paper), and red on TCCS input data. The 'Gurchiek' and 'Day' methods did not have coefficients, weights, or biases fit to individual coordinate systems and showed systematically higher errors in the WCS.
